# Trends in Non-invasive Prenatal Screening and Invasive Testing in Denmark (2000–2019) and Israel (2011–2019)

**DOI:** 10.3389/fmed.2021.768997

**Published:** 2021-11-17

**Authors:** Lena Sagi-Dain, Amihood Singer, Olav B. Petersen, Stina Lou, Ida Vogel

**Affiliations:** ^1^Prenatal Genetics, Genetics Institute, Carmel Medical Center, Affiliated to the Ruth and Bruce Rappaport Faculty of Medicine, Technion - Israel Institute of Technology, Haifa, Israel; ^2^Community Genetics, Public Health Services, Ministry of Health, Jerusalem, Israel; ^3^Department of Obstetrics, Center for Fetal Medicine, Copenhagen University Hospital Rigshospitalet, Copenhagen, Denmark; ^4^Department of Clinical Medicine, University of Copenhagen, Copenhagen, Denmark; ^5^Center for Fetal Diagnostics, Aarhus University, Aarhus, Denmark; ^6^Department of Clinical Genetics, Aarhus University Hospital, Aarhus, Denmark

**Keywords:** chromosomal microarray analysis, chromosome aberration, prenatal testing, non-invasive prenatal screening, invasive testing

## Abstract

**Introduction:** Following the wide distribution of non-invasive prenatal genetic screening (NIPS), numerous studies have reported a decline in total invasive tests in the recent years, up to 50–70% in some countries. However, in Denmark and Israel we have not experienced these declines. The objective of our study was to evaluate the trends in NIPS and chromosomal microarray analysis (CMA) use in Denmark and Israel.

**Methods:** This retrospective study was performed by data acquisition from the Danish Cytogenetics Central Registry throughout the years 2000–2019, and Israeli Public Health Services, Ministry of Health computerized database (from 2011).

**Results:** Of the 1,243,956 live births registered in Denmark over the years 2000–2019, a relatively steady level of invasive testing around 6% was noted since 2004, as opposed to 13.0% in Israel based on 1,594,962 live births between 2011 and 2019. The average uptake of NIPS was 1.1 ± 0.5% in Denmark vs. 4.3% in Israel (2013–2019). Relatively steady rates of invasive testing were noted in both countries, compared to a slight decline in NIPS in the recent years.

**Discussion:** The recent decrease in the rates of invasive testing in the NIPS era was not observed in Denmark or in Israel. These results imply that Danish and Israeli women and/or health providers might favor the high resolution and yield of CMA testing over the non-invasiveness of NIPS. We explore and discuss this phenomenon, based on five central factors.

## Key Message

The recent decrease in the rates of invasive testing in the NIPS era was not observed in Denmark or in Israel. These results imply that Danish and Israeli women and/or health providers might favor the high resolution and yield of CMA testing over the non-invasiveness of NIPS.

## Introduction

Following decades-long stability, in the recent years prenatal screening and diagnostic protocols are undergoing rapid changes. In contrast to previous relatively universal setups, in which women of age were offered invasive testing and karyotyping, diverse national strategies have evolved. These changes have been propelled by the development and application of technologies such as chromosomal microarray analysis (CMA) and Non-Invasive Prenatal Screening (NIPS); however, these advances alone do not explain the heterogeneous implementation and outcomes in different countries. In Holland and Belgium, for example, NIPS is first line tier, as the non-invasive risk-free testing has become the priority. On the contrary, in Denmark and Israel combined first trimester testing (cFTS) has been maintained. In the cFTS, a risk assessment is calculated through a combination of serum free beta human chorionic gonadotropin, pregnancy-associated plasma protein A along with maternal age and nuchal translucency measurement.

Since Israel and Denmark are very different countries in terms of population size and ethnic diversity, religious status, fertility rates etc., the use of similar prenatal screening setups in these places is intriguing. Thus, the aim of this paper is to compare our national data on prenatal testing and NIPS performed in recent years, and to suggest several factors that should be taken into consideration when aiming to understand the differences in current worldwide prenatal screening setups.

## Methods

### Denmark

**Figure 1:**
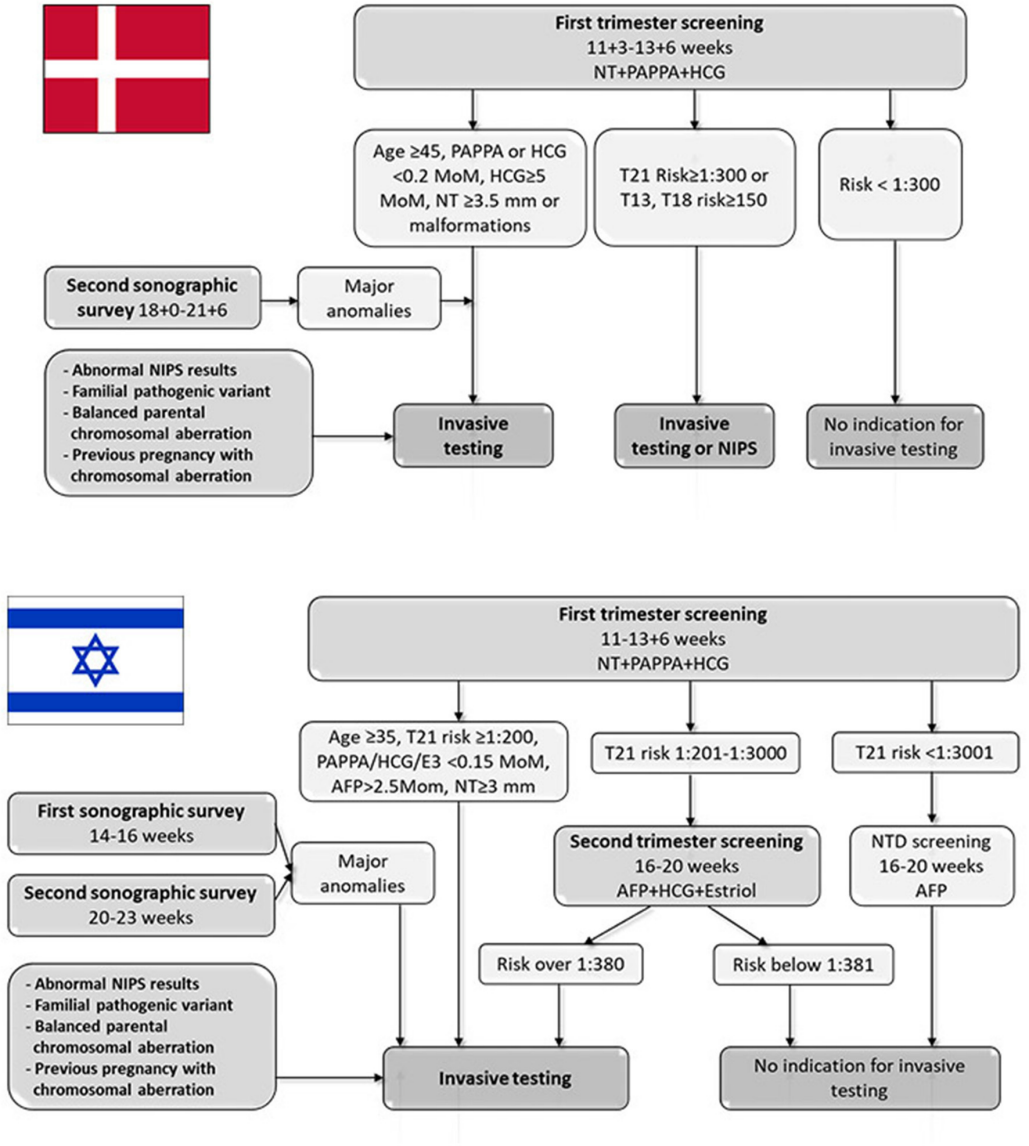
Danish and Israeli algorithms of national screening program for identifying pregnancies at risk for chromosomal aberrations. AFP, Alpha-fetoprotein; HCG, human chorionic gonadotrophin; NIPS, non-invasive prenatal screening; NT, nuchal translucency; NTD, neural tube defects; PAPPA, Pregnancy-associated plasma protein A.

Since 2004, all pregnant women in Denmark have been offered free-for-all, tax-financed cFTS at weeks 11+3–13+6, as well as a second trimester ultrasound screening for fetal anomalies at weeks 18+0–21+6 (Figure 1). The cFTS includes risk assessment for the common trisomies, performed according to the Fetal Medicine Foundation algorithm using maternal factors (age, body mass index, diabetes, smoking, conception status, PAPP-A, free beta HCG), and nuchal translucency measurement. Over 90% of the women in Denmark opt-in for cFTS.

Since January 2017, revised national guidelines on prenatal screening and diagnosis have included NIPS as an alternative to invasive testing to women at increased risk of chromosomal anomalies (Figure 1). The guidelines emphasize that NIPS should only be offered as an alternative to invasive testing to a limited group of women at high risk after cFTS defined as ≥1:300 for Trisomy 21 or ≥ 1:150 for Trisomies 18 or 13 (1). In case of fetal malformations or a single outlier of the four cFTS risk parameters (maternal age ≥45 years, PAPP-A <0.2 MoM, free beta HCG <0.2 or ≥5 MoM or nuchal translucency measurement ≥3.5 mm), the woman will be recommended to undergo invasive testing with CMA. Women receiving abnormal NIPS results are offered genetic counseling and invasive testing, also free of charge.

NIPS is also offered at private clinics in Denmark, at a cost of ~750$, which is not reimbursed by the state. This is still used relatively infrequently in Denmark, compared to other countries (2).

TOP is a legal right for all women up to week 11+6. Thereafter it must be approved by a regional council. This procedure operates under a gradual approach—the more severe the fetal disorder, the higher is the accepted gestational week for termination. TOP is not approved after the limit of viability (gestational age of 22+6 weeks).

Since 1960, all cytogenetic pre- and postnatal results are annually reported by all genetic laboratories to the Danish Cytogenetics Central Registry. The prenatal data are validated with postnatal or abortion data by the data manager. The NIPS platforms used by Danish public laboratories were a shallow whole genome sequencing approach including software analysis tools validated solely for trisomies 21, 18, and 13. Gender is also usually reported. Over the years 2015–2019 CMA has gradually replaced karyotyping for all prenatal indications for invasive samples. Data from 2000 and 2019 were retrieved from The Danish Cytogenetics Central Registry regarding both NIPS and invasive testing.

### Israel

In 2019, Israeli population consisted of 9,021,000 citizens, 74.2% of them are of Jewish origin and 20.9% are Arabs. The Jewish ultra-orthodox community comprises about 10% of Israeli population (3), while the Arab population is mostly Muslim (17.4%) (4). Israel has the highest fertility rates of all Organization for Economic Cooperation and Development countries−3.1 children per woman (5).

The Israeli National screening program for identifying women at risk for Down syndrome program includes several components (Figure 1), all free of charge and covered by the health maintenance organizations (HMO) or the Ministry of Health (MOH). The various testing and screening options are mostly presented by the attending primary care obstetrician. cFTS is the recommended test for all pregnant women, covered by the HMO since 2013. The uptake of this test by the pregnant population is about 60–70%. Women with nuchal translucency of 3 mm and above are referred to genetic counseling and advised to receive invasive testing for CMA analysis, covered by the HMO. A similar recommendation is given to women with high-risk results of first trimester screening tests (over 1:200). Pregnancies with an intermediate risk (1:201–1:3000) at cFTS are referred to integrated testing (i.e., combination of first and second trimester screening). Low-risk women (risk above 1:3001 on first trimester screening) are advised to perform only the alpha-fetoprotein (AFP) measurement as part of neural tube defects screening. Women with combined risk over 1:380 according to second trimester triple screening (AFP, HCG and unconjugated estriol) or integrated test are eligible for amniocentesis for fetal chromosome analysis, also covered by the HMOs. In addition, invasive testing for fetal chromosome analysis is free of charge for women older than 35 years at the onset of pregnancy regardless of other indications, covered by the MOH. Finally, invasive testing is covered by the MOH in cases of major sonographic anomalies or relevant family history, such as parental balanced chromosomal rearrangement, carrier couples for autosomal recessive diseases, or previous pregnancy with chromosomal aberration. From 2013, CMA costs for the abovementioned indications were covered by the MOH. For other indications, including personal wish, privately paid CMA testing was an option (for ~750$). From 2019 CMA is the routine test performed to any woman who undergo invasive test during pregnancy. The non-indicated invasive tests due to maternal request are common in Israel and constitute about 20–25% of the annual tests. Finally, a mid-trimester sonographic anatomic survey is performed at two target time points (around 15 and 22 weeks), both covered by the HMO.

NIPS has been available in Israel since 2013 and is currently offered by several private companies and one public hospital. A national position paper regarding the use of NIPS in Israel was published in 2014 and updated in 2018 (6). An option to perform NIPS is routinely offered and explained by obstetricians and genetic counselors, with an emphasis that this is a screening and not diagnostic modality. All women with increased risk at conventional Down screening tests are referred to genetic counseling and receive a recommendation to perform invasive testing by CMA. Thus, currently NIPS is neither included in the algorithm of recommended screening tests, nor as a substitute for invasive testing in case of abnormal maternal serum screening. All NIPS tests are aimed at the common trisomies and sex chromosome aneuploidy, while most of the companies also offer testing of common copy number variants (CNVs) (7). The price of basic NIPS testing in Israel approximates 750$, with a reimbursement of up to 75% of the cost depending on the HMO complementary health insurance policy. Abnormal NIPS results must be assured by invasive testing (following genetic counseling, covered by the HMOs).

TOP in Israel is allowed at any gestational age, following a mandatory approval of a specialized committee, with strict criteria after 24 weeks of gestational age for termination approval (mainly genetic or structural anomalies).

Retrospective data acquisition for this study was performed using Ministry of Health computerized database, which includes yearly numbers and proportions of invasive analyses, and the corresponding numbers and proportions of NIPS tests (registered since 2013).

### Ethical Approval

Since the data did not include any identifying details, the study was considered exempt of ethical approval.

## Results

Throughout the years 2000–2019, 1,243,956 live births were registered in Denmark—on average, 62,198 ± 3,168 births per year. Invasive testing was performed in 7.1 ±2.0% of the pregnancies (ranging from as high as 11.2% in 2000 prior to the initiation of the cFTS program in 2004 to a level around 6% after 2004). The average uptake of NIPS testing during the years 2013–2019 was 1.1 ± 0.5%, ranging from 0.2 to 3.3%.

In Israel, 1,594,962 live births were registered over the years 2011–2019 (8), averaging 177,218 ± 6,375 births per year. Of these, 13.0 ± 1.5% of the pregnancies underwent invasive prenatal testing, while NIPS was performed in 4.3 ± 0.7% of the cases.

**Figure 2:**
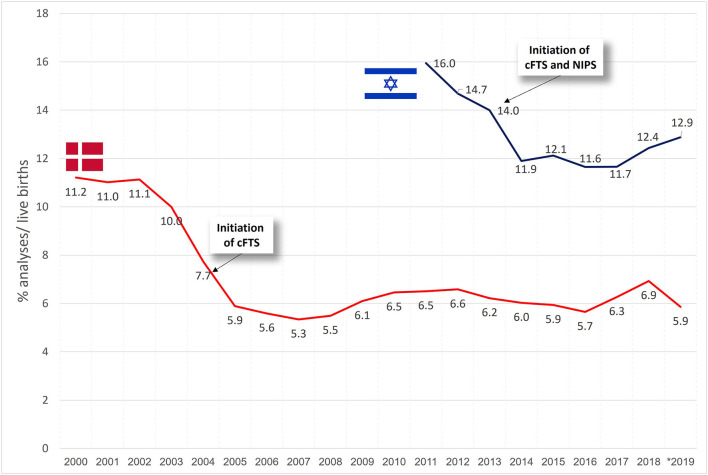
Trends in the uptake of invasive tests in % of live births in Denmark and Israel.

Figure 2 presents the trends in invasive testing per year. In Denmark, a decline in invasive testing was noted from 2003 and onwards (in conjunction with the introduction of cFTS in 2004), followed by a minimally varying rate of about 6% of the total pregnancies per year. In Israel, a decline was noted through 2011–2013 (of note, in 2011 the MOH stopped funding invasive tests for eligible women who performed it privately, while cFTS and NIPS were both introduced in 2013), showing relatively steady rates of invasive testing afterwards (~12%), with a slight increase to 12.4–12.9% during 2018–2019.

**Figure 3:**
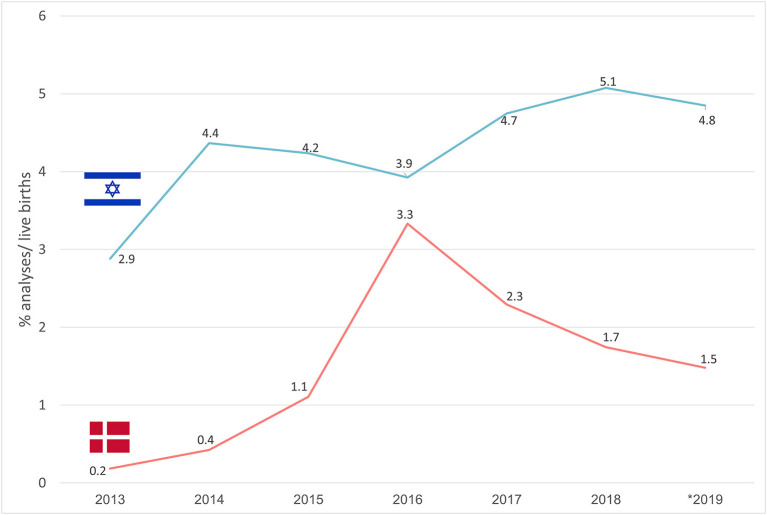
Trends in the uptake of NIPS in % of live births in Denmark and Israel.

In Figure 3 trends in the uptake of NIPS are described. In Denmark, a sharp increase in NIPS use was noted from 2013 (0.2%) to 2016 (3.3%), with a subsequent gradual decrease to 1.5% in 2019. In Israel lower variability in NIPS uptake was noted, reaching 5.1% in 2018 with a slight subsequent decline to 4.8% in 2019.

## Discussion

Our results show that both in Denmark and Israel, the national invasive rates have remained unchanged following the introduction of NIPS. In both countries, NIPS is used by <5% of the population, and its use seems to be further decreasing. This is in in contrast to numerous multi-center and population-based studies that have reported a decline in total invasive tests in “NIPS era,” up to 50–70% in some countries (9–11). Below, we explore and discuss this phenomenon, based on five central factors.

1) The availability of combined first trimester screening. cFTS is a well-established practice in many countries and has been successfully and universally implemented with high uptakes in Denmark and Israel for years. From a clinical perspective, the cFTS has advantages beyond the 85% sensitivity for the detection of Down syndrome (12), as the algorithm has also shown to be an effective indicator for other fetal diseases. For instance, first trimester sonography can detect up to half of fetuses with major or lethal structural anomalies (13). Early diagnosis also enables TOP by outpatient dilatation and curettage, which is offered up to 14 weeks in Denmark, and up to 18 weeks in most Israeli medical centers. Furthermore, first trimester ultrasound is important for correct dating, detection of multiple gestations or non-viable pregnancies. Thus, in Denmark and Israel there are perceived additional advantages of the cFTS, that cannot be achieved if NIPS was the only option for prenatal screening, as has been the case in some countries. NIPS is still expensive and focuses only on few syndromes; but may in time be included in and improve cFTS.

2) The perceived harms and benefits of the increased resolution by CMA over karyotyping or polymerase chain reaction aimed at common chromosomal aberrations. In both Israel and Denmark, the increased diagnostic yield of CMA has been demonstrated and reported by health professionals (14–16). CMA detection rate in pregnancies with malformations is about 8–12% and estimated as 4–8% in fetuses with increased nuchal translucency or with increased cFTS risk (15, 17). In low-risk cases, constituting the majority of pregnancies, CMA can detect clinically significant findings in 1:180 (18) and up to 1.4% (i.e., 1:71) (16). This number is lower but still impressive in women younger than 35 years-−0.71% or 1:130 (16). Interestingly, these risks are quite high relative to the risk of 1:300–1:380 for trisomy 21, a cutoff for which there have been survey programs and a recommendation to consider an invasive test for many years. In addition, it has been shown by predictive models that the 40% decrease in diagnostic procedures noted in US in the NIPS era would have missed 30,000 abnormal copy number variants and 4,000 standard aneuploidies only in the US population, resulting in potential costs of about $7 billion per year per 1 million patients for the healthcare system (19, 20). Nevertheless, the increased resolution of CMA also results in the identification of lower penetrance susceptibility loci, variants of unknown significance and late onset diseases. The potential clinical and individual consequences of such findings continue to be discussed, and medical societies in different countries may arrive to different conclusions. With CMA comes the need for more detailed pre- and post-test counseling, whereas NIPS provides more limited and specific information, and the preferences of the patients as well as the clinicians may vary. The 2-fold higher uptake of invasive testing in Israel (13.0 vs. 7.1% in Denmark) can partly be explained by the national genetic carrier-screening program able to detect carrier couples for numerous monogenic disorders (21), as well as the national next-generation-sequencing clinical program to identify the genetic cause in postnatal cases (for specific medical indications), allowing couples to avoid a similar case in the next pregnancy by performing an invasive test or pre-gestational testing. These indications account for about 5% of the total invasive tests per year. In addition, the previously described “quest for the perfect baby,” a common phenomenon in Israeli women, may explain the considerable rates of non-indicated invasive tests (22).

3) The perceived value of non-invasive, risk free screening opportunities for selected trisomies. NIPS was first introduced in Hong Kong in 2011 (23), and is currently offered in over 90 countries. Its major advantages are greater accuracy compared to cFTS for trisomy 21, 13 and 18, in conjunction with lack of complications and discomfort associated with invasive testing. In a high-risk pregnant population, sensitivity of NIPS reaches 99.8% for trisomy 21, 97.7% for trisomy 18, and 97.5% for trisomy 13, with a pooled specificity of 99.9% (24). The high sensitivity and specificity of the cell-free DNA test for several common trisomies has led many health practitioners as well as the public to consider NIPS an almost perfect substitute for invasive testing (25). This may explain the decrease in invasive testing in many countries. The reasons why NIPS has not gained popularity in Denmark and Israel compared to most other countries are unclear. We speculate that health practitioners in both countries might have an increased focus on prenatal genetic disorders beyond common trisomies. Israel has developed advanced carrier screening programs to reduce disease burden of pregnancy (21) while in Denmark, the screening guidelines refer to “target fetal disorders” in general rather than specifying on trisomies 13, 18 and 21. Possibly, this has made NIPS a less optimal choice in both Israel and Denmark, focusing on the fact that even the most advanced cell-free DNA screening techniques can at best present a rough estimation of fetal karyotype (26–28). In addition, this says something about how pregnant women are dependent on information when making decisions and will often follow the recommendations of professionals.

4) The acknowledgment of evidence pointing toward a lower than previously believed risk of miscarriage caused by invasive testing. In Denmark, new evidence that procedure-related risk of miscarriage is 0.1–0.2%was central to the decision of the Fetal Medicine Society to recommend invasive testing over NIPS. Also, in Israel health professionals openly prioritize invasive testing over NIPS. Our results show that invasive testing was performed in 13% of the pregnancies in Israel (i.e., 1 in every seven pregnancies), a much higher rate compared to 6% in Denmark, which again is higher than many other developed countries (i.e., 4.1% in Australia, 2% in USA, and <1% in the UK or the Netherlands) (9, 29–31) This finding by itself requires a separate research, and might explained by competition between local HMOs, as well as the emerging social pressure for comprehensive prenatal screening as an indispensable part of a good motherhood and future parental responsibility (22).

Finally, 5) The timing of the result availability and the possibilities for TOP. NIPS can be performed as early as 9–10 weeks of pregnancy, allowing earlier detection of anomalous pregnancies (32)—an issue of particular value in countries with limited access to pregnancy termination at later gestational ages. In Denmark, 80% of invasive tests have been chorionic villi samples (CVS) since the introduction of cFTS. This is probably driven by the possibility to receive a diagnostic result 3 weeks earlier using CVS compared to amniocentesis. Therefore, there is a great focus to obtain a diagnosis early in pregnancy to maintain reproductive choices. In Israel, more than 90% of invasive tests are amniotic fluid samples, and TOP is allowed until term. This, along with routine 15 weeks sonographic anatomic survey, may explain the tendency toward amniocentesis instead of CVS. This ought to give NIPS some popularity in Denmark, as diagnosis can be made early in pregnancy. We do, however, see that NIPS is often used by women not willing to risk invasive testing (33), and in particular we have identified an increase in children born with prenatally diagnosed Down syndrome. The total annual number of children born with Down syndrome has not increased, but rather the offer of NIPS now allows parents who do not want TOP but would like to be prepared for the birth of a Down syndrome baby to have a risk-free testing opportunity. We conclude that the earlier diagnosis by NIPS compared to CVS has not had substantial impact in Denmark (33).

Additional factor to explain the relatively low uptake rates of NIPS in Denmark and Israel might be the specific national guidelines with minimal inclusion of NIPS. However, previous evidence reporting decreased rates of invasive testing in the NIPS era was derived from countries with roughly similar guidelines at the time of data collection. For instance, a study of Larion et al. describing a total of 15,418 prenatal tests over the years 2004–2013 in Norfolk, Virginia, has noted a decline of 46.7% in amniocenteses following introduction of NIPS. In this period, NIPS was neither included in the national guidelines as a funded first-tier screening method, nor as an option to substitute invasive testing, similarly to Israeli prenatal management (9). Similarly, a large-scale population based Australian study by Hui et al. have described a 39.6% decline in invasive testing in the NIPS era (2013–2015), although NIPS was not subsidized by any government or private health insurance (11). Thus, non-inclusion of NIPS in national guidelines and funding seems not to be the main factor affecting its uptake in Denmark and Israel.

Our analysis has several limitations, the prominent of which is the lack of detailed description of all indications and baseline characteristics for the invasive testing. In addition, the extrapolation of various rates is based on yearly numbers of live births and not the number of overall pregnancies.

Despite differences in organization of health care, culture etc, our data nevertheless indicate that Israeli and Danish women prefer high-resolution and comprehensive CMA testing, a finding possibly influenced by new information provided by health care professionals about lower risk of miscarriage following invasive testing.

We interpret that women are responsive to new information and national guidelines about test performance and procedure-related risks—and maybe also the preferences of the health care personnel—though costs may also influence choices. We suggest that a variety of prenatal screening and testing methodologies should be available to all women, including the option of CMA or NIPS.

## Data Availability Statement

The original contributions presented in the study are included in the article/supplementary material, further inquiries can be directed to the corresponding author/s.

## Ethics Statement

Ethical review and approval was not required for the study on human participants in accordance with the local legislation and institutional requirements. Written informed consent for participation was not required for this study in accordance with the national legislation and the institutional requirements.

## Author Contributions

All authors have substantially contributed to the conception or design of the work, acquisition, analysis and interpretation of data for the work, drafting the manuscript or revising the manuscript critically for important intellectual content, and final approval of the version to be published and agree to be accountable for all aspects of the work in ensuring that questions related to the accuracy or integrity of any part of the work are appropriately investigated and resolved.

## Funding

IV holds a grant from the Novo Nordisk Foundation NNF16OC0018772. OP holds a professorship funded by Novo Nordisk Foundation grant NNFSA170030576.

## Conflict of Interest

The authors declare that the research was conducted in the absence of any commercial or financial relationships that could be construed as a potential conflict of interest.

## Publisher's Note

All claims expressed in this article are solely those of the authors and do not necessarily represent those of their affiliated organizations, or those of the publisher, the editors and the reviewers. Any product that may be evaluated in this article, or claim that may be made by its manufacturer, is not guaranteed or endorsed by the publisher.

## Abbreviations

CMA: Chromosomal Microarray Analysis
cFTS: combined first trimester screening
CVS: chorionic villus sampling
HMO: health maintenance organizations
MOH: Ministry of Health
NIPS: non-invasive prenatal testing.
